# Notes and News

**Published:** 1890-12-13

**Authors:** 


### Notes and News.

Collected and Communicated.

A case at the West London Police Court demands the attention of philanthropists; it is the old story of alleged sham firemen calling for subscriptions at suburban houses. The Charity Organisation Society has done much good work in exposing sham firemen, and surely the lesson of caution in such cases should have been learnt by now.

A deputation from the Committee of the Morley House Convalescent Home, near Dover, on Saturday afternoon waited upon the Lord Mayor, at the Mansion House, to ask his sympathy and support for an extension of the present home. Mr. H. M. Hamilton Hoare introduced the deputation, which numbered between forty and fifty persons, and said the workmen present had come to ask his lordship to help them in the matter of extension of the home. The Lord Mayor promised all the help in his power.

A fatal case of "sleeping sickness" has lately occurred in one of the London hospitals, and rumour states that there is some hope that the ever-present "microbe" in connection with this disease has been found. The man was a nigger, brought to England by Mr. Grattan Guinness, and he slept himself to death, all efforts to arouse him being absolutely futile. Luckily the "nona" scare is now over, so that public fears are not likely to be raised by this strange case, which is of the greatest interest to all medical men.

Leeds Infirmary has altered its management; an assistant ophthalmic surgeon has been appointed, and various other changes made. Under the old *régime*—which ruled that the election committee should consist of twelve governors, not members of the Weekly Board, two members of the Weekly Board, and the treasurer—lay members could have practically no voice in the election of the staff. The lay members of the Board had control of the entire staff, and yet they had no voice in their selection. To remedy this anomaly it was now proposed that the Special Committee, instead of consisting of fifteen, consist of twenty-five—that was twelve members elected as previously, twelve lay members, and the treasurer.

The *Daily News* is responsible for the following story:—Without pretending to rival Dr. Koch or M. Pasteur, Mr. Justice Hawkins has established a fair claim to a place among the discoverers of cures. At the Chelmsford Assizes last week a prosecutor had begun to give his evidence in half-inaudible tones, when this judge, with that twinkle in the eye which is so familiar to gentlemen at the bar, observed "I will give you something for your throat. Remember I shall not allow you any expenses if you don't speak louder." This prescription is stated to have been at once attended by a marvellous cure.

Glasgow Maternity Hospital issues as usual an excellent report, in which it proudly claims to stand second to no maternity hospital in the land. The medical report showed that at the institution there had been 387 indoor cases and 93 operative cases, while there had only been 5 deaths. The outdoor cases attended from the hospital numbered 1,456, and the operative cases 95, while the maternal deaths in the department also had only been 5. At the West-end Branch the number of women attended at their own homes had been 410. The operative cases were 26. There was only one maternal death there. After training and examination 37 nurses had received the diploma of the hospital. The treasurer's statement showed that the receipts, including the capital, had been £2,857 4s. 9d., and the expenditure £1,854 3s. 2d., made up of £1,723 10s. 11d., on the hospital and £130 12s. 3d., on the West-end Branch. The capital fund, which included a salary of £100, was thus £1,003 1s. 7d.

There is no lack of *savoir faire* about the bright little Cottage Hospital at Boscombe. It, therefore, goes without much saying that the bazaar got up in aid of its greatly-needed funds should be a huge success. Under distinguished patronage, it was opened by the Mayor of Bournemouth, in all the pristine freshness of his new honours. The first ceremony by the first Mayor of the famous watering-place was performed with all suitable dignity. Flowers and music galore enlivened the scene. All sorts of pretty things decorated the stalls—not the least being the fair faces of the vendors, set off by the ever-attractive nursing uniform—and buyers went away well satisfied with their bargains. To the popular matron, Miss Thomassen, who bore the heaviest share of the undertaking, the greatest credit is due, and will be heartily accorded by all. The result of the bazaar is, we understand, a sum of £250, which will be handed over to the Cottage Hospital.

On December 4th, the Court of Contributors (a much more euphonious title than the usual Court of Governors) to the Glasgow Victoria Infirmary was held, and an excellent report presented. Since the last annual meeting, the erection and furnishing of the first section of the infirmary buildings have been successfully completed. The buildings erected comprise the administrative block for the entire institution when fully completed, and one pavilion, containing three large and three small wards, with the necessary adjuncts. They also comprise washing-house and laundry, boiler and engine-house, mortuary, and entrance lodge. Like the administrative block, these buildings will suffice for the infirmary when completed. The whole of the grounds have also been laid off and walled in. The ordinary income has amounted to £2,889 5s. 6d., of which no less than £592 1s. 5d., or more than one-fifth, has been contributed by employés in public works, &c. The ordinary expenditure has amounted to £2,341 0s. 3d., leaving a balance of £548 5s. 5d. to be carried forward to next year. For a full year's expenditure it is anticipated that £4,000 will be required. Nearly 500 in-patients have been treated, and 2,114 out-patients.

THE following vacancies are announced this week, the amount of the salary in each case being given after the name of the institution :—

**Resident Medical Officers.**

Royal Free Hospital, Junior Medical Officer—Board, &c.
Victoria Hospital for Children, House Physician—£50, board, &c.
Denbigh Infirmary, House Surgeon—£85, board, &c.
Morpeth Dispensary, House Surgeon—£120, rooms, &c.
Beverley Asylum, Assistant Medical Officer—£100, board, &c.
Carmarthen Infirmary, House Surgeon—£100, board, &c.
Matlock Hydropathic, Junior Physician—£200, board, &c.

**Non-Resident Medical Officers.**

Lincoln Oddfellows' Institute, Assistant Medical Officer—£104.
Royal Free Hospital, Registrar.
City of London Lying-in Hospital, District Surgeons.

ONE last appeal to our readers to remember how numerous the adults in the hospital wards are. If any of our friends have not seen our former appeals we explain once more that we desire parcels of clothing for distribution at Christmas to adult patients. The children who are in hospital at the festive season are well cared for by *Truth*, the Post Office employés, and others; and it is rather sad to see the thin-faced men and women looking on only when the Christmas tree is in progress. A warm petticoat or pair of socks would be such a charming present to them just when recovering from illness and going forth to work again. Parcels or gifts of money will be received up till December 17th, and should be addressed "Christmas Parcel, Hospitals' Association, Norfolk House, Norfolk Street, Strand, London." We acknowledge with thanks parcels from Nurse Simpson, of Mildmay, N. N. N., Yeovil, Somerset, and from Millie and Lena.

THE little Madeira Seamen's Hospital owes it origin to the energy and generosity of Mrs. Gordon Duff, Dr. Julius Goldschmidt, and Mrs. James Bottomley, and it was first established six years ago in the town of Funchal. It has treated on an average about twenty-six patients per annum, and in one respect differs from most hospitals where free patients are received in that a patient's friend or relation is allowed to reside in the hospital on paying for board and lodging. There are eight beds, and the building stands in its own grounds, which are laid out as a flower and kitchen garden. One room is utilised as a library. A fair amount of support is accorded to the institution by residents and visitors, and by others interested in Madeira. The matron is Miss H. Van Schermbeck, and she is ably supported by Mrs. Mary Lamster as nurse. The success of this little hospital is to no small extent due to the interest taken in it by Messrs. Blandy Bros. and Co., 16, Mark Lane, and Madeira, who are its hon. treasurers.

IN support of the Royal Free Hospital, the second annual grand ball is announced to take place on December 2nd, at the Holborn Town Hall. Looking at the long list of patrons secured on the present occasion, we may fairly regard this event as a regular fixture in the future. The names include, amongst others, Sir Julian Goldsmid, Bart., M.P., Sir Alexander E. Miller, Q.C., J.P., D.C.L., Alderman Sir F. Wyatt Truscott, Mr. Robert Grant Webster, M.P., Mr. H. L. W. Lawson, M.P., Mr. T. H. Bolton, M.P., Mr. Gainsford Bruce, Q.C., M.P., Captain F. T. Penton, M.P., Mr. W. W. B. Beach, M.P., Lieutenant-General Gordon Pritchard, C.B., R.E., Mr. Sheriff Augustus Harris, Mr. Henry Oakley, general manager of the Great Northern Railway, as well as Mr. E. Ford North, chairman, and several other members of the Weekly Hospital Board. There about fifty gentlemen on the ball committee, the hon. treasurer being Mr. J. T. Balls, of 362, Gray's Inn Road, and the hon. secretary, Mr. Walter H. Brinklow. With so many active advocates the success of the entertainment should be assured.

MR. BRANDRETH, the other day, at a public meeting held at Kensington Town Hall for the purpose of making known the objects and aims of the Charity Organisation Society, speaking of beggars, said that in Kensington, alas, mendicancy was too profitable to be abandoned when once taken up. As a ruined working man once remarked to a friend of their committee, he did not know before how easy it was, and he was not going back to work again. It was no trouble to walk sixty streets before midday, and it must be an odd street where he did not get one penny. That was 30s. a-week, and all the afternoon to himself. One public provided the 5s. in the morning and the other too often received it before night. If the inhabitants of Kensington would only refrain from giving in the streets, without enquiry, for six months, there would be no beggars left in that neighbourhood.

ON Wednesday evening, at the Middlesex Hospital, Mr. Rathbone, M.P., in the chair, Miss Wilson read, before the Hospitals' Association, an interesting paper on "Nursing in Workhouses and Workhouse Infirmaries." Miss Wilson commenced with a reference to the good work done by the late Dr. Benton, and then went on to tell some tales, which she subsequently used to point morals. For instance, in one infirmary it was the custom of the matron and master to go round with the medical officer. Miss Wilson forcibly pointed out the necessity for trained matrons. The following is an abstract of the duties of a matron in a large London infirmary, containing nearly seven hundred beds, thirty-six nurses, and a few probationers: "She is expected to enforce order and cleanliness in the infirmary, to cause the paupers on their admission to be properly cleansed and clothed, to superintend the making of clothes and linen, to take charge of all crockery and other utensils, to give the necessary directions for the drying and getting up of linen, and to report to the medical officer any negligence and misconduct on the part of assistants or servants." No nursing qualification is mentioned, and no arrangement made for the direct control of the nursing staffs; the legal definition of the post may, in fact, be defined as that of a housekeeper. Contrast with these inadequate provisions for the control and efficiency of the nursing staff, the first rule for the matron at the Birmingham Infirmary: "To govern and control the nurses, and the sick in the wards, under the supervision of the medical staff; to be responsible for the cleanliness, punctuality, and good order of the nurses and of the inmates, and for the proper condition of the wards and of the nurses' home; to report direct to the Committee at their ordinary meetings."

MISS WILSON summed up the necessary reforms as follows: "In Metropolitan Infirmaries: Medical Superintendent and the election of a trained matron, and a clear definition of her duties in relation to the nursing staff, by an alteration of the present 'orders.' The appointment of trained charge nurses, and the engagement of a larger number of probationers, who, in return for a thorough training, would be expected to remain in the infirmary for a definite period. In Large Provincial Infirmaries: The complete separation of the sick from the workhouse; the engagement of a larger number of nurses, especially for night duty; the appointment of a resident doctor and of a trained superintendent of nurses. In Country Workhouses: The increase of the nursing staff, and the consequent decrease of pauper help; the subordination of the nurses, in matters relating to the sick, to the medical officer, in place of the master and matron. To carry out these, or any other remedial measures, it is necessary that a healthy public opinion should be promoted, which, in time, would raise the level of intelligence amongst guardians. In conclusion, the gist of the whole matter, and indeed the principle that must underlie any detailed statement, is, that the sick and the able-bodied require distinct treatment, and, therefore, separate organization; and that no great advance can be made in the matter of nursing in workhouse infirmaries until this fundamental principle obtains clear recognition." There was an interesting discussion, which we hope to give next week.

